# Analysis of polychlorinated alkanes in food by liquid chromatography–tandem mass spectrometry

**DOI:** 10.1007/s00216-025-06070-0

**Published:** 2025-08-21

**Authors:** Ingus Perkons, Laura Lazdina, Dzintars Zacs

**Affiliations:** 1 Institute of Food Safety, Animal Health and Environment “BIOR”, Riga, 1076 Latvia; 2 University of Latvia, Riga, 1004 Latvia

**Keywords:** Polychlorinated alkanes, Chlorinated paraffins, Tandem mass spectrometry, Liquid chromatography, Persistent organic pollutants, Food safety

## Abstract

Polychlorinated alkanes (PCAs), the principal constituents of chlorinated-paraffin technical mixtures, are persistent, bioaccumulative pollutants that raise growing toxicological concern. Due to their complexity, PCA analysis in food remains analytically challenging, predominantly relying on high-resolution mass spectrometry applications. This study aimed to develop and validate a more accessible liquid chromatography–tandem mass spectrometry (LC–MS/MS) method for quantifying PCA-C_10–17_ in food commodities. Six reversed-phase columns were evaluated during the study, and phenyl-hexyl and biphenyl stationary phases provided superior separation of critical isobaric PCA homologues. Ammonium acetate (5 mM) was used as a mobile phase additive to promote the formation of acetate adducts, enhancing selectivity in MS/MS settings by minimizing the impact of deprotonated species on product-ion spectra. Validation experiments conducted using fortified samples demonstrated satisfactory recoveries (PCA-C_10–13_, 88%; PCA-C_14–17_, 121%; and PCA-C_10–17_, 103%). Comparative analyses using six interlaboratory test materials and a certified fish matrix reference material confirmed the method’s accuracy. All *z*-scores for PCA-C_10–13_ were ≤|2|, and only 2 results for PCA-C_14–17_ were in the questionable range (|*z*|= 2–3). In the certified reference material, measured values for PCA-C_10–13_ were within the certified range, while those for PCA-C_14–17_ were near its lower boundary. The developed method was compared to the conventional high-resolution mass spectrometry, showing a strong agreement between the results of both instrumental setups. These results establish this LC–MS/MS protocol as an accessible and reliable alternative to PCA monitoring within food safety and regulatory frameworks.

**Graphical Abstract:**

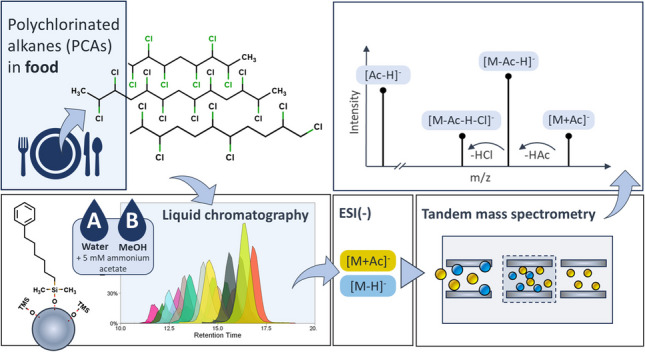

**Supplementary Information:**

The online version contains supplementary material available at 10.1007/s00216-025-06070-0.

## Introduction

Polychlorinated *n*-alkanes (PCAs; not to be confused with principal component analysis) are complex and persistent chemical compounds resistant to chemical and biological changes. They are the main component of chlorinated paraffin (CP) technical mixtures used in various industrial applications such as plasticizers, flame retardants, and lubricants. PCAs have a general formula of C_*x*_H_2*x*+2−*y*_Cl_*y*_, in which the carbon chain length typically varies between 10 and 30 atoms and the chlorine content is 30–70% by weight. They are historically grouped by their carbon chain length into three groups: PCA-C_10–13_, PCA-C_14–17_, and PCA-C_18–30_, formerly referred to as short-chain CP (SCCP), medium-chain CP (MCCP), and long-chain CP (LCCP), respectively [1, 2].

Due to their persistence and the potential for bioaccumulation and biomagnification, PCAs are found ubiquitously in environmental samples such as air, water, and soil [3, 4], as well as in the tissues of living organisms [5–7] and various food products [8–11]. According to the recent Norwegian cohort study by Yuan et al. [12], diet is regarded as the primary exposure route for PCA intake in humans [12]. However, the recent risk assessment of PCAs by the European Food Safety Authority (EFSA) was inconclusive, partly because of insufficient occurrence data [11].

Reports suggest that long-term exposure to PCAs, particularly PCA-C_10–13_, could have adverse health effects on humans. Among these, oxidative stress and disruptions in energy metabolism are considered the primary concerns [13]. Hence, PCA-C_10–13_ have been scrutinized over recent years and were categorized as persistent organic pollutants (POPs) in Annex A of the Stockholm Convention in 2017. This decision led the industry to substitute short-chain PCAs with PCAs-C_14–17_ and PCAs-C_18–30_. However, in 2025, due to similar toxicity profiles, PCAs-C_14–17_ were also added to Annex A of the Stockholm Convention (with specific exemptions) at the 12th meeting of the Conference of the Parties (COP‑12) [14].

PCAs are typically analyzed using gas or liquid chromatography coupled to mass spectrometry (GC–MS or LC–MS). The vast complexity of PCAs makes their instrumental analysis and quantification challenging. According to Vetter et al. [1], the number of plausible isomers within PCA-C_10–13_ is approximately 42,500, while for PCA-C_14–17_, it could reach up to a million. As a result, complete chromatographic separation is impossible. Moreover, under electrospray ionization (ESI) conditions typically used in LC-based approaches, PCAs can form several types of ions, most commonly adduct ions such as [M + Cl]^−^ and [M + Ac]^−^ (with chloride or acetate, respectively), depending on the mobile phase composition. Deprotonated species ([M–H]^−^) are also formed to a lesser extent, further complicating the resulting mass spectra. Furthermore, even under ideal ionization conditions, where each PCA congener produces exactly one type of ion per molecular species, frequent overlaps of isobaric ions with similar abundances are inevitable for congeners differing by five carbon and two chlorine atoms [15]. Consequently, most current PCA analysis methods rely on high-resolution mass spectrometry (HRMS) as it can deliver comprehensive PCA analysis in a single run and, in some cases, even eliminate the need for chromatographic separation [16, 17]. While HRMS is a powerful technique and has become the gold standard in this field, it is also expensive and less accessible. Balancing cost and accuracy is often critical, particularly in resource-limited settings or for routine monitoring or screening purposes. For such cases, liquid chromatography–tandem mass spectrometry (LC–MS/MS) can be an attractive alternative. However, the number of PCA analysis methods using LC–MS/MS reported in the literature is minimal. Matsukami et al. [18] employed LC–MS/MS to study PCA-C_10–13_ in mixed plastic wastes, and McGrath et al. [19] adapted this method to analyze PCA-C_10–17_ in rubber consumer products and toys. Yet, to the authors’ knowledge, no studies have utilized LC–MS/MS to analyze PCAs in food samples, leaving a significant gap in current methodologies. This shortfall may stem from unsatisfactory fragmentation behavior of PCAs or mass interferences that hinder sensitivity, preventing trace-level PCA analysis.

Therefore, the aim of this study was to develop and validate an LC–ESI–MS/MS method for the quantitative determination of PCA-C_10–17_ in food commodities, with the goal of offering a feasible and accessible alternative to high-resolution mass spectrometry techniques. The method was specifically designed to address key analytical challenges, including the chromatographic separation of isobaric congeners and limited ionization efficiency under low-resolution MS conditions. An additional objective was to evaluate the method’s suitability for routine food analysis by assessing its performance across spiked food samples, interlaboratory test materials, and a certified reference material.

## Experimental

### Chemicals and materials

Pesticide grade *n*-hexane and dichloromethane, as well as HPLC grade acetonitrile and methanol, were purchased from Honeywell Riedel-de Haën™. Ultrapure deionized water (18 MΩ∙cm) was produced by a Milli-Q water purification system (Millipore, Bedford, MA, USA). Ammonium chloride and ammonium acetate were acquired from Acros Organics (Morris Plains, NJ, USA). Sulfuric acid (95.0–97.0%) was purchased from Honeywell FLUKA. Anhydrous sodium sulfate, Florisil® (0.150–0.250 mm), and silica gel (70–230 mesh) were obtained from Merck. The sorbents were heated for 12 h at 600 °C before use and stored in airtight glass bottles. Acidic silica was prepared by adding concentrated sulfuric acid to silica gel to achieve a final acid content of 44% (w/w). Florisil was deactivated with water to a moisture content of 2% (w/w).

Single-chain standard mixtures for PCA-C_10–13_ homologue groups and internal standard (ISTD) ^13^C_10_-labeled 1,5,5,6,6,10-hexachlorodecane were purchased from Dr. Ehrenstorfer (Augsburg, Germany). Single-chain standard mixtures for PCA-C_14–17_ homologue groups were synthesized at the University of Hohenheim and kindly provided by the European Union Reference Laboratory for Halogenated Persistent Organic Pollutants in Feed and Food (EURL POPs). The complete list of standards and their composition in calibration mixtures is provided in Supplementary Table 1.

Six analytical columns were used in this study: Kinetex C18 (100 × 2.1 mm, 2.7 µm), Kinetex Phenyl-Hexyl (100 × 2.1 mm, 2.6 µm), Kinetex Biphenyl (100 × 2.1 mm, 2.6 µm), Kinetex PAH (100 × 2.1 mm, 3.5 µm), Luna PFP(2) (100 × 2 mm, 3 µm), and Synergi Max-RP (150 × 3.0 mm, 3 µm). All columns were obtained from Phenomenex (Torrance, CA, USA).

Lard and coconut fat interlaboratory study test materials were provided by the EURL POPs (Freiburg, Germany). Matrix reference material (ERM®-CE100) prepared from wild wels catfish (*Silurus glanis*) was purchased from the Joint Research Centre, European Commission (Geel, Belgium).

### Sample preparation

The sample preparation protocol was adapted from Perkons et al. [20] with minor modifications. For wet samples, 2.5–5 g was transferred into a 50-ml polypropylene centrifuge tube and dried by adding anhydrous sodium sulfate. Subsequently, 100 µl of internal standard (1,5,5,6,6,10-hexachlorodecane, 500 pg/µl) was added, and the sample was extracted twice using 25 ml of a *n*-hexane/dichloromethane (1:1, v/v) mixture. The combined extracts were transferred to a clean 50-ml tube. For lipid-rich samples such as fats and oils, the drying step with Na_2_SO_4_ was omitted. Instead, 1 g of sample was directly placed into a 50-ml PP tube and diluted with 30 ml of *n*-hexane/dichloromethane (1:1, v/v) before acid treatment. Acid treatment was performed by adding 6 ml of concentrated sulfuric acid to the extract. The mixture was vigorously shaken and centrifuged for 10 min at 3500 rpm. The resulting organic layer was transferred to a heart-shaped flask, evaporated using a rotary evaporator, and reconstituted in approximately 1 ml of *n*-hexane. The extract was then subjected to clean-up using a glass column packed with three sorbent layers: Florisil (3 g, bottom), silica gel (3 g, middle), and acidic silica gel (4 g, top). The column was preconditioned with 20 ml of *n*-hexane/dichloromethane (1:1, v/v) followed by 20 ml of *n*-hexane. After the sample application, the column was flushed with 40 ml of *n*-hexane. After that, PCAs were eluted with 70 ml of *n*-hexane/dichloromethane (1:1, v/v) and collected in a 100 ml heart-shaped flask. The eluted fraction was evaporated in a rotary evaporator until the remaining volume was around 1 ml. This extract was transferred to a 2-ml vial, gently evaporated under a nitrogen stream, and solvent exchanged to 50 µl of methanol.

### LC–MS/MS method

Chromatographic separation was carried out on a Dionex UltiMate 3000 UHPLC system (Thermo Fisher Scientific, San Jose, CA, USA) using a Kinetex Phenyl-Hexyl column (100 × 2.1 mm, 2.6 µm). A binary gradient system was used, comprising water (mobile phase A) and methanol (mobile phase B), both containing 5 mM ammonium acetate. The flow rate was maintained at 0.25 ml/min. The column compartment was set to 35 °C, and the sample tray was kept at 14 °C. The gradient program was as follows: 50% B from 0 to 1 min, increased to 75% B from 1 to 2 min, then gradually increased from 75 to 95% B between 2 and 13 min using a concave gradient profile, and further raised to 100% B from 13 to 14 min. This composition was held isocratically until 18.0 min, followed by a return to the initial condition (50% B), which was maintained until the end of the run at 22.0 min. The injection volume was 5 µl.

Detection was performed using a TSQ Altis MS/MS system (Thermo Fisher Scientific, San Jose, CA, USA) operating in negative ESI mode. The main source parameters were as follows: spray voltage, 3500 V; sheath gas (N_2_), 50 arbitrary units (a.u.); auxiliary gas (N_2_), 10 a.u.; sweep gas (N_2_), 1 a.u.; ion transfer tube temperature, 325 °C; and vaporizer temperature, 350 °C. The collision gas pressure was set to 1.5 mTorr, and all measurements were carried out in selected reaction monitoring (SRM) mode. Two SRM transitions were selected for each PCA homologue group, with a dwell time of 200 ms per SRM scan. A full list of SRM transitions and corresponding collision energies (CE) is provided in Table 1. Instrument control and data processing was done in TSQ Altis Tune Application 3.4 and Thermo Scientific Xcalibur 4.7, respectively. More detailed information about the instrumental parameters used throughout the optimization experiments is outlined in Supplementary Sect. 1.

**Table 1:** SRM transitions, retention times, and collision energies for PCA homologue groups analyzed by LC–MS/MS

PCA homologue group	RT (min)	SRM 1, m/z (CE, V)	SRM 2, m/z (CE, V)
C_10_H_17_Cl_5_	11.3	373- > 313 (5)	373- > 59 (10)
C_10_H_16_Cl_6_	11.7	407- > 347 (5)	407- > 59 (12.5)
C_10_H_15_Cl_7_	12.3	441- > 381 (5)	441- > 59 (12.5)
C_10_H_14_Cl_8_	12.5	477- > 417 (5)	477- > 59 (12.5)
^13^C_10_H_16_Cl_6_ (ISTD)	12.7	417- > 59 (7.5)	419- > 59 (7.5)
C_11_H_19_Cl_5_	12.3	387- > 327 (5)	387- > 59 (10)
C_11_H_18_Cl_6_	12.5	421- > 361 (5)	421- > 59 (12.5)
C_11_H_17_Cl_7_	12.9	455- > 395 (5)	455- > 59 (15)
C_11_H_16_Cl_8_	13.1	491- > 431 (7.5)	491- > 59 (15)
C_12_H_21_Cl_5_	12.5	401- > 341 (5)	401- > 59 (10)
C_12_H_20_Cl_6_	13.3	435- > 375 (5)	435- > 59 (14)
C_12_H_19_Cl_7_	13.5	469- > 409 (7.5)	469- > 59 (15)
C_12_H_18_Cl_8_	13.7	505- > 445 (7.5)	505- > 59 (15)
C_13_H_23_Cl_5_	13.6	415- > 355 (5)	415- > 59 (12.5)
C_13_H_22_Cl_6_	13.8	449- > 389 (5)	449- > 59 (15)
C_13_H_21_Cl_7_	14.8	483- > 423 (7.5)	483- > 59 (15)
C_13_H_20_Cl_8_	14.9	519- > 459 (7.5)	519- > 423 (10)
C_14_H_25_Cl_5_	14.9	429- > 369 (5)	429- > 59 (14)
C_14_H_24_Cl_6_	15.5	463- > 403 (7.5)	463- > 59 (15)
C_14_H_23_Cl_7_	15.5	497- > 437 (7.5)	497- > 59 (15)
C_14_H_22_Cl_8_	15.7	533- > 473 (7.5)	533- > 437 (10)
C_15_H_27_Cl_5_	15.7	443- > 383 (5)	443- > 59 (12.5)
C_15_H_26_Cl_6_	15.8	477- > 417 (5)	477- > 59 (12.5)
C_15_H_25_Cl_7_	16.0	511- > 451 (7.5)	511- > 59 (15)
C_15_H_24_Cl_8_	16.1	547- > 487 (7.5)	547- > 451 (10)
C_16_H_29_Cl_5_	16.4	457- > 397 (5)	457- > 59 (10)
C_16_H_28_Cl_6_	16.4	491- > 431 (7.5)	491- > 59 (15)
C_16_H_27_Cl_7_	16.5	525- > 465 (7.5)	525- > 59 (15)
C_16_H_26_Cl_8_	16.7	561- > 501 (7.5)	561- > 465 (10)
C_17_H_31_Cl_5_	16.8	471- > 411 (7.5)	471- > 59 (12.5)
C_17_H_30_Cl_6_	17.0	505- > 445 (7.5)	505- > 59 (15)
C_17_H_29_Cl_7_	17.2	539- > 479 (7.5)	539- > 59 (15)
C_17_H_28_Cl_8_	17.2	575- > 515 (10)	575- > 479 (10)

### LC-HRMS method

The HRMS methodology followed the protocol established in our previous study [21], which provides detailed information on chromatographic separation and data post-processing. In the present study, the only modification was the use of a different HRMS instrument—Q Exactive Orbitrap MS (Thermo Fisher Scientific, San Jose, CA, USA). The mass spectrometer operated in negative ESI mode with a resolution of 140,000 (at m/z 200) and a scan range of m/z 150–1000. The spray voltage was set to 2500 V. The capillary temperature was 256 °C, and the auxiliary gas heater was 413 °C. The latter values were set based on the vendor software’s built-in algorithm that considers the flow rate for adjusting source temperatures. Nitrogen was used as the nebulizer, sheath, and auxiliary gas, with sheath and auxiliary gas flow rates of 48 a.u. and 11 a.u., respectively. Data acquisition was done using Thermo Scientific Xcalibur 4.1 software.

### Quantification

Quantification was performed according to the approach described in Perkons et al. [21], which is based on the method proposed by Reth et al. [22]. Briefly, peak areas of individual PCA homologues were normalized to the internal standard and corrected using procedural blanks. Homologues were grouped by carbon chain length, and their relative abundances were used to estimate the chlorine content within each group. Calibration was conducted using four CP mixtures at multiple concentration levels to derive chlorine content–dependent response factors. Extrapolation beyond the calibration range was avoided. Instead, the nearest experimentally determined response factor was applied. For HRMS method, quantification was based on the peak area of the most abundant isotopologue of the [M + Cl]^−^ species for each PCA homologue group, whereas for MS/MS analysis, peak areas of the primary SRM transition for [M + Ac]^−^ species were used, as listed in Table 1.

## Results and discussion

### Selection of the stationary phase and optimization of LC gradient conditions

When low-resolution MS/MS detection is employed for detecting PCAs, isobaric interferences are far more likely to happen compared to HRMS-based methods. Hence, efficient LC separation plays a crucial role in minimizing these interferences. For example, when considering the isotopologue signals formed from [M + Cl]^−^ adducts of PCA-C_10–17_Cl_4–9_ congeners with intensities above a 10% threshold, it is evident that in several cases, the most abundant isotopologue for each PCA congener overlaps with at least one other isotopologue from a different congener, as the Δm/z values are less than 1 Da. The most intense overlaps occur because of congeners with a chain of five carbons longer but containing two fewer chlorine atoms (see Supplementary Fig. 1). A notable example can be observed for C_10_H_13_Cl_9_, which produces the most abundant signal at m/z 486.78. It can suffer from 3 interferences from other PCA congeners: ^12^C_12_^1^H_18_^35^Cl_4_^37^Cl_5_ (m/z 486.84; 11%), ^12^C_15_^1^H_25_^35^Cl_7_^37^Cl (m/z 486.94; 89%), and ^12^C_17_^1^H_30_^35^Cl_3_^37^Cl_4_ (m/z 487.00; 11%). Moreover, this example is just the tip of the iceberg. Additional spectral interferences may also be present, including co-eluting matrix compounds, other PCA adducts, and other ingredients of CP mixtures such as chlorinated olefins and diolefins [16, 23]. Nevertheless, in this study, the focus was placed on the most critical and frequent isobaric overlaps. Consequently, the selection of the stationary phase and gradient optimization targeted the separation of key PCA pairs: C_*n*_H_2*n*+2−*m*_Cl_*m*_ and C_*n*+5_H_2(*n*+5)−*m*_Cl_*m*−2_.

Matsukami et al. [18] and Amoura et al. [24] have already studied PCA behavior under different LC stationary phases. Matsukami et al. [18] evaluated columns with cyanopropylsilane (CN) and C18 stationary phases for PCA-C_10–13_, ultimately selecting the CN column for the final method. This decision was based on its superior peak separation compared to the C18 column, enabling complete resolution between PCA-C_*n*_ and PCA-C_*n*+3_ chain-length congener groups [18]. Meanwhile, Amoura et al. [24] evaluated six LC stationary phases (C18, C30, CN, porous graphitic carbon, pentafluorophenyl, and phenyl-hexyl) to determine the most suitable approach for the LC separation of PCA-C_10–20_. Among the tested columns, the C30 stationary phase demonstrated the best performance, likely due to enhanced hydrophobic interactions. In contrast, CN and porous graphitic carbon columns yielded incomplete homologue group patterns, as the most highly chlorinated PCAs were often undetected [24]. In this study, we evaluated six stationary phases: regular C18, higher-bonding density C18 (PAH), C12 (Synergi Max-RP), phenyl-hexyl, biphenyl, and pentafluorophenyl (PFP). During preliminary testing, each column was assessed by injecting a mixture of PCA-C_10–17_ using nine different gradient programs. Details of these gradient programs are provided in Supplementary Table 2.

**Fig. 1:**
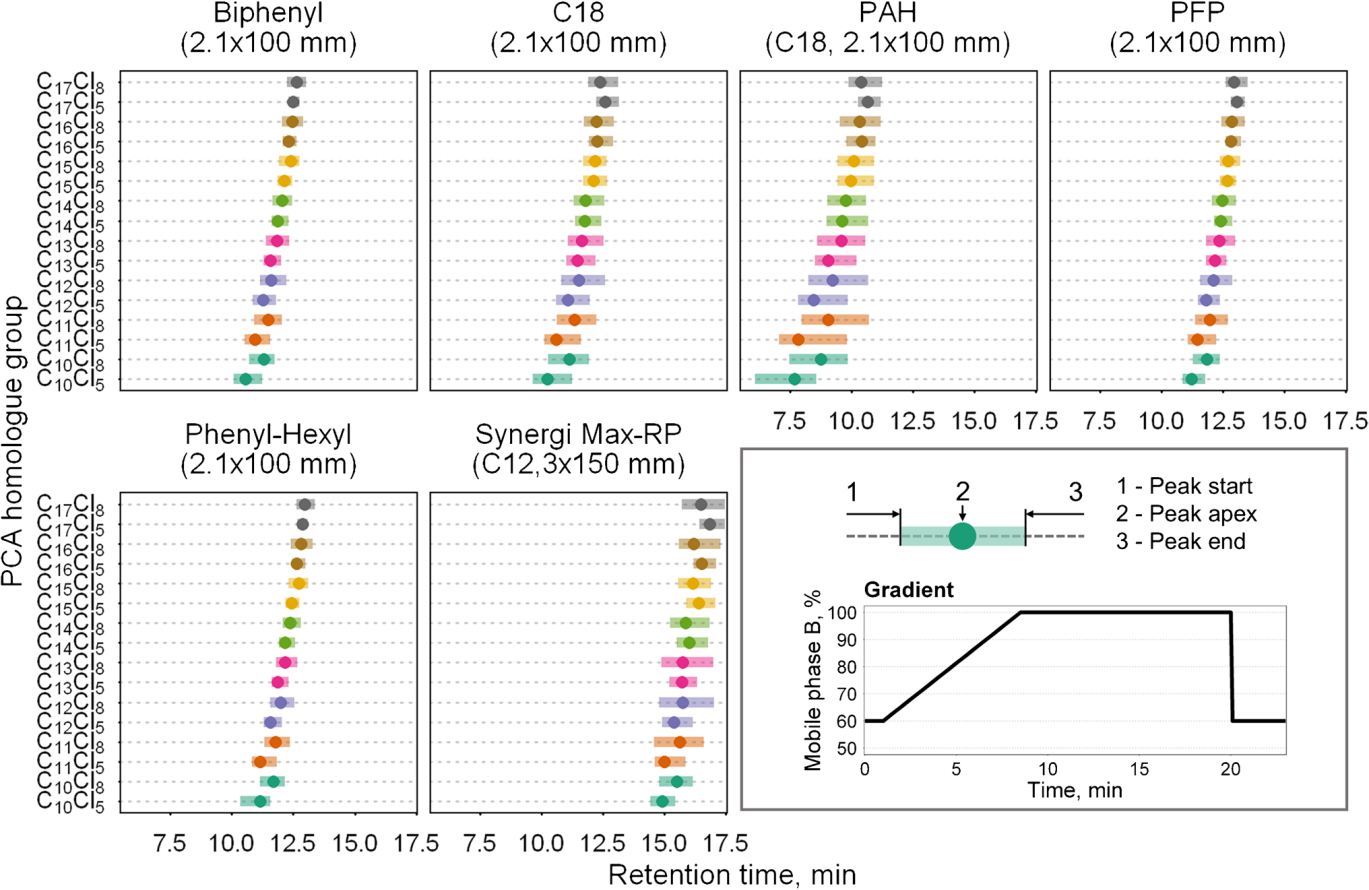
Retention of PCA-C_10–17_Cl_5_ and PCA-C_10–17_Cl_8_ homologue groups on various stationary phases

Preliminary testing of the stationary phases revealed that the overall retention mechanism remained almost consistent across all phases tested. As illustrated in Fig. 1, the retention order was primarily determined by chain length. However, increased peak width was observed for the PAH and Synergi Max-RP columns, likely due to larger particle size, which can lead to less efficient mass transfer and increased band broadening.

Interestingly, a change in retention order was noted for longer-chain PCAs with higher degrees of chlorination in all instances except the biphenyl and phenyl-hexyl phases. For example, PCA-C_16_Cl_8_ and PCA-C_17_Cl_8_ eluted before PCA-C_16_Cl_5_ and PCA-C_17_Cl_5_, respectively. This phenomenon could be explained by steric effects caused by vicinal chlorine atoms positioned along the *n*-alkane backbone. These chlorine atoms create steric hindrance with the stationary phase, weakening hydrophobic interactions and causing the more highly chlorinated homologues to elute earlier than their less chlorinated counterparts with the same chain length. A similar trend has been observed in the calculation of octanol–water partition coefficients (log *K*_OW_). For instance, the log *K*_OW_ value for 1,1,1,3,9,11,11,11-octachloroundecane is approximately 7.2, while for 1,1,1,3,6,7,10,11-octachloroundecane, it is 5.4. This difference directly results from chlorine atom placement: the former configurational isomer has five consecutive carbon atoms without chlorine substituents, making it more hydrophobic, whereas the latter has only two such carbons, reducing its hydrophobicity [25]. Although the PFP stationary phase was expected to enhance the retention of highly chlorinated PCAs through dipole–dipole interactions, the electron-withdrawing effect of chlorine atoms on PCAs appeared to have little influence on their retention order.

Given that a change in retention order for longer-chain PCAs could negatively impact the LC–MS/MS method’s performance by increasing overlap among later-eluting homologue groups, only the biphenyl and phenyl-hexyl columns were selected for further method development. In the second optimization stage, both columns were evaluated under isocratic conditions at 60%, 70%, 80%, and 90% mobile phase B to determine the composition at which each PCA chain length becomes unretained on the stationary phase. Supplementary Fig. 2 shows that PCA-C_10–13_ eluted when the organic phase reached 80%, whereas PCA-C_14–17_ required 90% MeOH for elution. These findings indicate that an appropriate gradient for PCA-C_10–17_ should fall within this range.

**Fig. 2:**
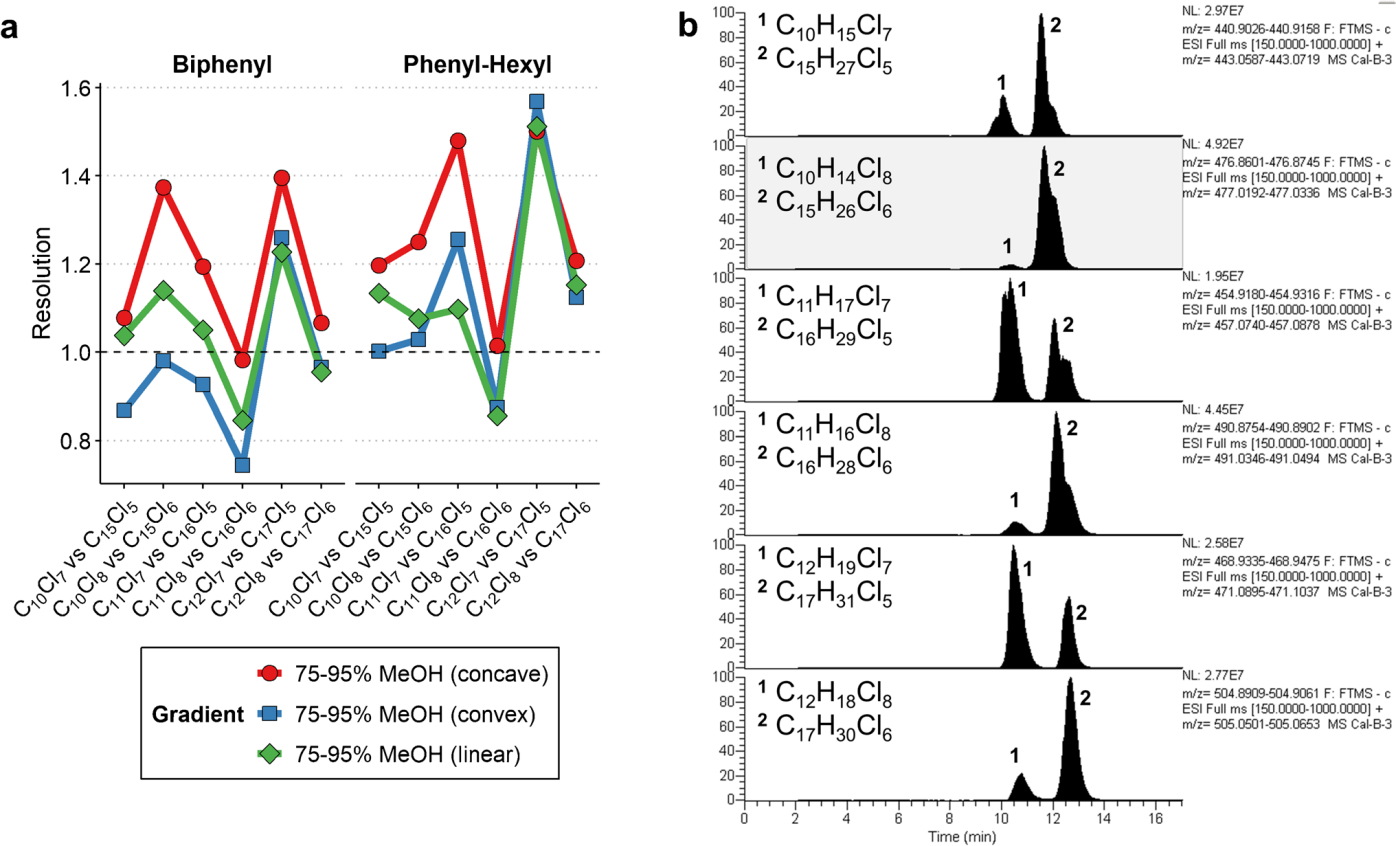
Peak resolution between critical PCA homologue pairs on biphenyl and phenyl-hexyl LC columns (**a**) and an example chromatogram of these PCA homologue pairs on a phenyl-hexyl column (**b**)

In the final LC optimization stage, three gradient types were tested, each differing only in the segment from 2 to 13 min, where mobile phase B increased from 75 to 95%. The first gradient employed a linear increase, while the other two followed concave and convex profiles (see Supplementary Fig. 3). To identify the most suitable gradient for PCA separation, a chromatographic peak resolution was calculated for the critical PCA pairs responsible for the most significant isobaric interferences in low-resolution MS. As shown in Fig. 2, the phenyl-hexyl column provided slightly better resolution values than the biphenyl column, and the concave gradient ensured a resolution > 1 for all critical PCA homologue group pairs. Hence, this combination was selected for the final method.

### Selection of mobile phase additive and MS/MS fragments

Efficient ionization of PCAs under negative ESI conditions requires adduct formation, as deprotonated [M − H]^−^ species provide poor sensitivity for low-chlorinated PCAs [2]. The most common approach is chloride enhancement, which promotes [M + Cl]^−^ adduct formation via direct addition of dichloromethane to the mobile phase [24], post-column introduction via a T-piece [26], or incorporation of chloride salts such as ammonium chloride or tetramethylammonium chloride [21, 27, 28]. Other adducts, such as [M + Ac]^−^ and [M + Br]^−^, can be formed using ammonium acetate [18, 19] or ammonium bromide [27, 29]. We focused solely on ammonium chloride and ammonium acetate as mobile phase additives to simplify PCA determination.

Regardless of the mobile phase additive used, deprotonated [M − H]^−^ species are expected to remain present. Their relative abundance decreases with increasing chain length and decreasing chlorination degree [24, 28], meaning that shorter-chain, highly chlorinated PCAs form less stable adducts and produce a greater proportion of [M − H]^−^ ions. Acetate adducts have been shown to be less stable than chloride adducts [28]. In our study, 5 mM ammonium acetate and 0.025 mM ammonium chloride in methanol were tested, with results aligning with previous findings. Peak area data for both mobile phases are presented in Supplementary Figs. 4 and 5. Acetate adducts showed lower stability, with [M − H]^−^ ions accounting for up to 40% of the total peak area for some PCAs (e.g., PCA-C_11_Cl_8–9_). In contrast, chloride adducts had lower [M − H]^−^ contribution, typically below 10% and not exceeding 25%. Source vaporizer temperature optimization did not significantly improve adduct stability and, hence, was left as the manufacturer’s default value for the set flow rate (350 °C for 0.3 ml/min).

**Fig. 3:**
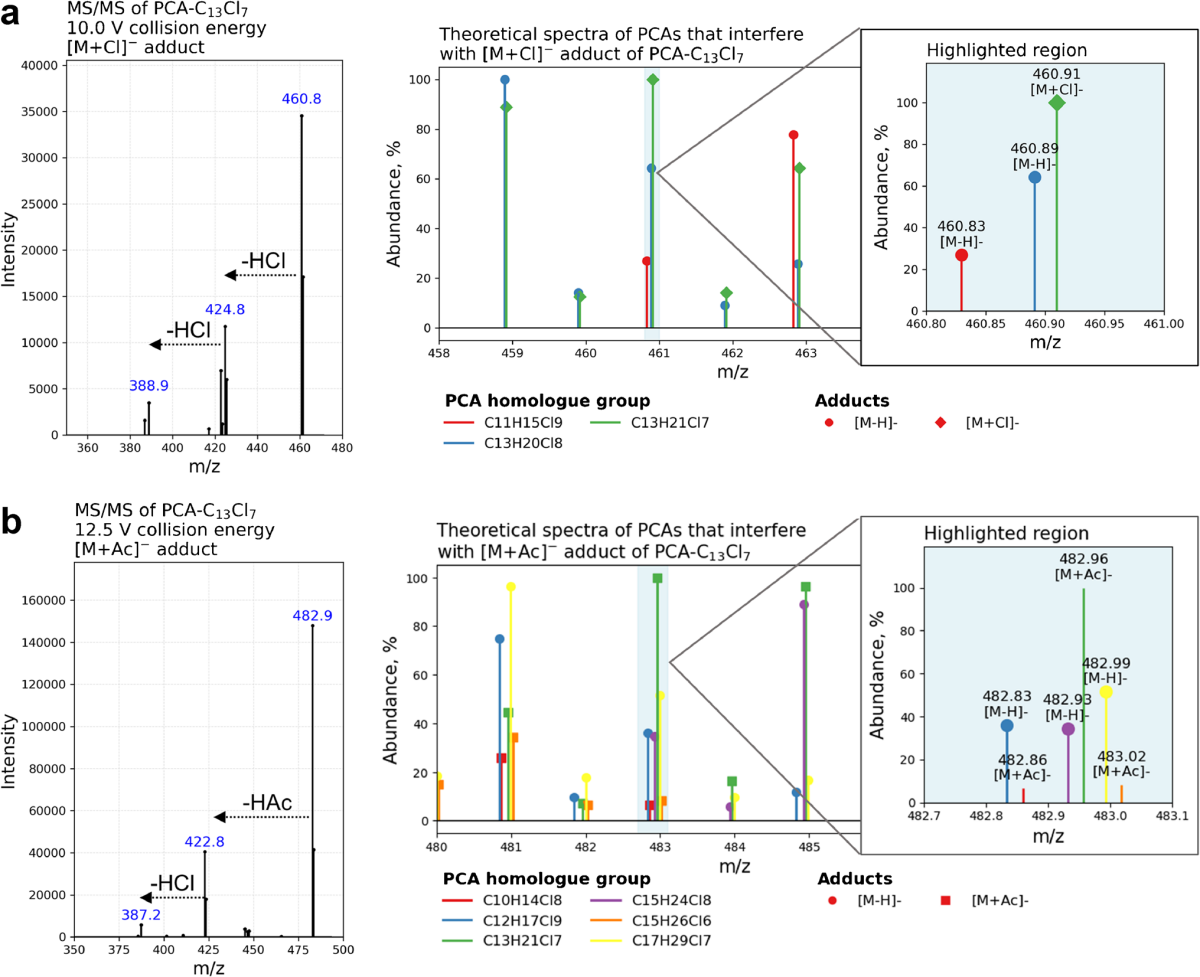
An example of fragmentation pattern for PCA-C_13_Cl_7_ chloride adducts (**a**) and acetate adducts (**b**) along with potential isobaric interferences present within a ± 0.5 m/z isolation window

The choice of adducts influences both the fragmentation pattern and the sensitivity and selectivity of the LC–MS/MS method. To examine these effects, we studied the fragmentation behavior of PCA-C_10–17_Cl_5–8_ using product ion scan mode at CE values ranging from 5 to 40 V. Optimization graphs are presented in Supplementary Fig. 6. Fragmentation of acetate [M + Ac]^−^ adducts began at just 5 V, with the primary loss being the acetic acid (CH_3_COOH, 60 Da), due to the acetate group, yielding the deprotonated molecule, [M − H]^−^. For PCAs with six or fewer chlorine atoms, the only other fragment observed was the deprotonated acetic acid at m/z 59. In contrast, for higher-chlorinated PCAs, additional fragments appeared, corresponding to sequential losses of 36 Da (HCl) and 72 Da (2 × HCl) from the [M − H]^−^ ion (see Fig. 3b). Yet, these fragments were of comparably low intensity and disappeared completely with increased CE values. These findings are in line with Matsukami et al. [18], who studied PCA-C_10–13_ via LC–MS/MS in mixed plastic waste and employed acetate-enhanced ionization [18].

The fragmentation behavior of chloride adducts was similar. The first fragment at low CE values was deprotonated species, indicating a loss of HCl, and additional HCl losses were observed only for higher-chlorination degree PCAs (see Fig. 3a). In addition, fragment ions at m/z 35 (^35^Cl^−^) and m/z 37 (^37^Cl^−^) were also present at higher CE values. In terms of absolute intensity, the acetate adducts were able to produce slightly more intense product ions. Contrary to general scenario, where the fragment intensities are somewhat comparable to the precursor initial intensity obtained in full-MS mode, a significant drop of response was observed for PCAs regardless of the adduct used. This indicated that during the fragmentation PCAs may undergo extensive neutral losses or secondary gas-phase reactions without generating abundant charged product ions.

Recognizing that isobaric interferences could significantly compromise the selectivity of the LC–MS/MS method, we evaluated which adduct would be more suitable for minimizing such effects. As shown in Fig. 3 (right panel), isobaric interferences were observed for both chloride and acetate adducts of PCA-C_13_Cl_7_. The [M + Cl]^−^ adduct (top right panel, Fig. 3a) overlaps with isotopologue signals from deprotonated C_13_H_20_Cl_8_ and C_11_H_15_Cl_9_ species, both of which produce identical fragment ions (i.e., ^35^Cl^−^, ^37^Cl^−^, and [M-H-HCl]^−^). Hence, MS/MS does not offer additional selectivity to differentiate between chlorine and deprotonated species.

In contrast, the [M + Ac]^−^ adduct (bottom right panel, Fig. 3b) overlaps with five isotopologue signals. Three arise from deprotonated species and two from acetate adducts of other PCAs. While this may appear substantial, the deprotonated species exhibit distinct fragmentation patterns compared to acetate adducts. They will not be seen in MS/MS mode, allowing for better LC–MS/MS analysis selectivity. Additionally, ammonium acetate is a commonly used volatile salt in LC–MS/MS, whereas ammonium chloride may decompose in the ESI source, potentially leaving non-volatile residues that could lead to excessive instrumental contamination. For these reasons, 5 mM ammonium acetate was selected for the final method.

During final method testing, we faced a challenge detecting the internal standard (^13^C-labeled PCA-C_10_Cl_6_). Quantifying PCAs requires numerous concurrent SRM scan events since we monitor four homologue groups (C_n_Cl_5–8_) for each chain length. As a result, the scan frequency was substantially reduced. For PCAs, this posed no issue, as their chromatographic peaks, approximately 1 min wide, were adequately sampled with scans every 2–3 s. However, the internal standard’s peak, less than 10 s wide, received insufficient scans to achieve the recommended 10 data points. To address this, we added a 20-s segment to the SRM function list, during which the MS/MS system exclusively scanned the internal standard (see Supplementary Fig. 7). During the validation experiments, we did not observe any substantial shift in retention time for the internal standard that could be induced by the presence of matrix. Although this modification created brief intervals with reduced data collection for some PCAs co-eluting with the internal standard, quantification accuracy remained largely unaffected because PCA peaks were broad enough to ensure reliable peak integration by seamlessly bridging data before and after these intervals.

### Method validation

An in-house validation was conducted by analyzing a series of fortified sunflower oil samples. Three fortification levels were assessed: level A (75 µg/kg PCA-C_10–13_ and 150 µg/kg PCA-C_14–17_), level B (150 µg/kg PCA-C_10–13_ and 75 µg/kg PCA-C_14–17_), and level C (100 µg/kg PCA-C_10–13_ and 100 µg/kg PCA-C_14–17_). Five replicates per level were examined on the same day. The validation results are shown in Table 2. The mean recoveries for PCA-C_10–13_, PCA-C_14–17_, and PCA-C_10–17_ were 88%, 121%, and 103%, respectively, with the coefficient of variation (CV) ranging from 7 to 13% across the 3 PCA homologue groups. The limit of quantification (LOQ) was determined using procedural blanks, calculated as the mean blank value plus three standard deviations across all measurements. The LOQs were 22 ng per vial for PCA-C_10–13_ and 88 ng per vial for PCA-C_14–17_. Due to the complexity and undefined isomeric composition of technical mixtures, we did not determine the limit of detection (LOD), as it would be highly congener- and matrix-dependent and difficult to interpret meaningfully. A more extensive multi-day validation was not performed, as the primary source of error in PCA analysis stems from discrepancies between PCA profiles in calibration and real samples. This is particularly relevant because the instrumental response is highly dependent on the specific isomeric composition of the PCA mixture. When fortified samples are prepared and quantified using the same technical mixture, the response factors (RFs) are inherently well matched, leading to artificially high precision and recovery estimates. Consequently, validation based solely on fortified samples may significantly overestimate the method’s true accuracy in real-world applications [30, 31]. To address this, we analyzed multiple interlaboratory test materials obtained from the EURL POPs (Freiburg, Germany).

**Table 2:** A summary of results from fortification experiments for the LC–MS/MS method

Parameter	Level A (75 µg/kg PCA-C_10–13_, 150 µg/kg PCA-C_14–17_, *n* = 5)	Level B (150 µg/kg PCA-C_10–13_, 75 µg/kg PCA-C_14–17_, *n* = 5)	Level C (100 µg/kg PCA-C_10–13_, 100 µg/kg PCA-C_14–17_, *n* = 5)	All levels
**PCA-C**_**10–13**_				
Recovery range (min–max, %)	82–99	78–85	85–101	78–101
Mean recovery (%)	91	81	93	88
Coefficient of variation (%)	7	3	6	7
**PCA-C**_**14–17**_				
Recovery range (min–max, %)	85–123	114–132	118–143	85–143
Mean recovery (%)	112	123	130	121
Coefficient of variation (%)	16	7	11	13
**PCA-C**_**10–17**_				
Recovery range (min–max, %)	84–114	90–100	101–118	84–118
Mean recovery (%)	105	95	111	103
Coefficient of variation (%)	12	4	7	11

In total, six interlaboratory materials were evaluated, each analyzed in triplicate. Additional details about test materials are provided in Supplementary Table 4. For PCA-C_10–13_, all *z*-scores across replicates (*n* = 15) were ≤|2|, indicating that the LC–MS/MS method produced satisfactory quantitative results. For PCA-C_14–17_, 2 out of 15 results showed questionable performance (2 <|*z*-score|< 3), while for the sum of PCAs, all results were satisfactory. A comprehensive list of measured results and calculated *z*-scores is presented in Supplementary Table 5.

Finally, the developed LC–MS/MS method was applied to analyze the newly available matrix reference material (ERM-CE100, fish fillet) from the Joint Research Centre of the European Commission (JRC, Belgium) [32]. This reference material was tested in three replicates. For PCA-C_10–13_, the results ranged from 26 to 34 µg/kg, falling within the certified range (31 ± 9 µg/kg). For PCA-C_14–17_, greater variation was observed, with measurements ranging from 27 to 53 µg/kg. One result was at the lower boundary of the certified value (44 ± 17 µg/kg) but remained acceptable. Full results are detailed in Supplementary Table 6.

In conclusion, the in-house validation and subsequent testing of the LC–MS/MS method demonstrated its applicability for quantifying PCA-C_10–13_ and PCA-C_14–17_ in food samples. While the method exhibited satisfactory precision and accuracy for PCA-C_10–13_ across all tested scenarios, some variability was observed for PCA-C_14–17_, particularly in the interlaboratory and reference material analyses, where a few results indicated questionable performance or borderline acceptability. Overall, the developed method offers a robust tool for PCA analysis, with potential for further refinement to improve consistency across diverse sample types and homologue groups.

### Comparison between LC–MS/MS and LC-HRMS methods

To further evaluate the applicability of the newly developed LC–MS/MS method for the analysis of PCAs in food samples, extracts from interlaboratory test materials and certified reference materials were re-analyzed using an Orbitrap Q-Exactive HRMS system. In addition, several real food samples previously shown to contain high levels of PCAs were included and analyzed by both methodologies.

**Fig. 4:**
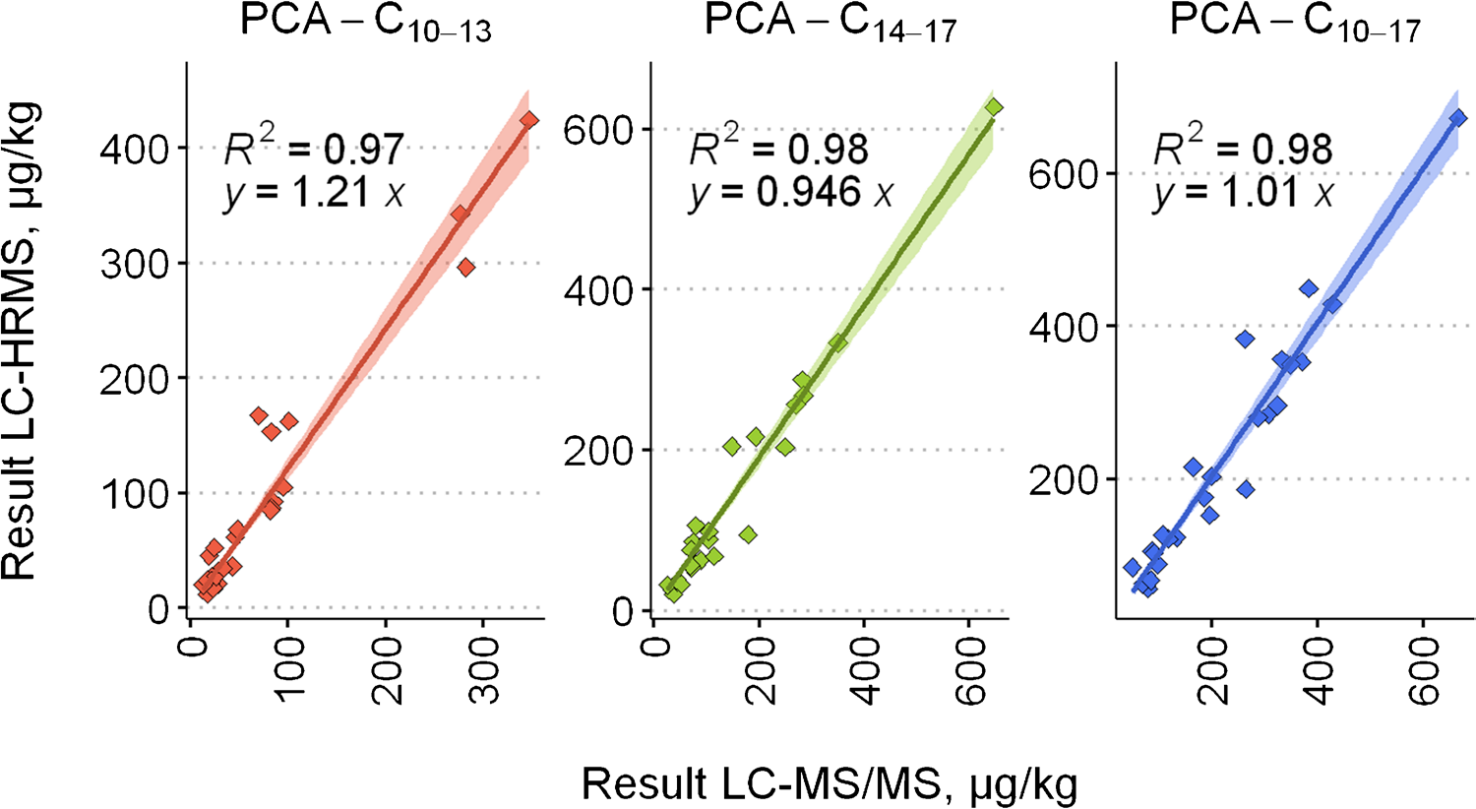
Linear regression of PCA concentrations obtained from the same sample extracts using LC–MS/MS and LC-HRMS methods

As shown in Fig. 4, the coefficients of determination (*R*^2^) indicate strong agreement between the datasets across all homologue groups, ranging from 0.97 to 0.98. However, it should be noted that the regression slope is influenced by a high-concentration leverage point, which may affect its reliability as an indicator of proportionality across the entire concentration range. The slope for PCA-C_10–13_ was 1.21, suggesting a systematic positive bias in the LC-HRMS measurements relative to LC–MS/MS. This may indicate that LC-HRMS results are slightly overestimated or, conversely, that LC–MS/MS results are slightly underestimated. Given the validation results, which showed a mean recovery of 88% for shorter-chain PCAs (Table 2), the latter explanation appears more plausible.

A major concern with the LC–MS/MS method was the potential for isobaric interferences leading to overestimated PCA concentrations. However, the comparison with HRMS data demonstrates that the LC–MS/MS method offers sufficient selectivity and systematic overestimation is absent. Moreover, our results indicate that, in line with the findings of McGrath et al. [33], a narrower selection of PCA homologues can still yield reliable quantitative results, provided that the most abundant chlorination degrees are adequately covered in the analytical method.

## Conclusions

In this study, we developed an LC–ESI–MS/MS method for the quantitative analysis of PCA-C_10–17_ in food samples. Among six tested LC columns, phenyl-hexyl and biphenyl stationary phases provided the most effective separation of critical isobaric PCA homologue pairs. Ammonium acetate was selected as the optimal mobile phase additive, enabling the formation of [M + Ac]^−^ adducts and offering improved selectivity in MS/MS settings compared to chloride-based ionization.

The method was validated using fortified food samples and further tested with interlaboratory test materials and a certified reference material. For PCA-C_10–13_, recoveries and repeatabilities were consistent across all test scenarios. Although slightly higher variability was observed for PCA-C_14–17_, the results remained within acceptable ranges. A comparative analysis with an Orbitrap-based LC-HRMS method showed strong correlation (*R*^2^ = 0.97–0.98), confirming the selectivity and reliability of the LC–MS/MS approach.

Given its accessibility and satisfactory performance, the developed LC–MS/MS method offers a practical alternative to high-resolution techniques, particularly for laboratories involved in routine monitoring of PCAs in food commodities. Further optimization may improve accuracy for longer-chain PCAs and broaden the method’s applicability to other complex matrices.

## Supplementary Information

Below is the link to the electronic supplementary material.

ESM 1 (DOCX 823 KB)

## Data Availability

The authors have included additional data in Supplementary Information, and further details are available upon request.
