# Supplementary material for: Analysis of polychlorinated alkanes in food by liquid chromatography–tandem mass spectrometry
Source: Anal Bioanal Chem. 2025 Aug 21;417(24):5533–44. doi: 10.1007/s00216-025-06070-0 (PMC12521293; doi:10.1007/s00216-025-06070-0)
Supplement: Supplementary file 1 — (DOCX 823 KB) [file 216_2025_6070_MOESM1_ESM.docx]

**Electronic Supplementary Information**

**Analysis of polychlorinated alkanes in food by liquid chromatography–tandem mass spectrometry**

Ingus Perkons^1*^, Laura Lazdina^1,2^, Dzintars Zacs^1^

^1^ Institute of Food Safety, Animal Health and Environment “BIOR”, Lejupes iela 3, LV-1076, Riga, Latvia

^2^ University of Latvia, Jelgavas iela 1, LV-1004, Riga, Latvia

* Corresponding author; e-mail address: [ingus.perkons@bior.lv](mailto:ingus.perkons@bior.lv)

**Table of Contents**

[S1. Summary of instrumental parameters for the optimization experiments 2](#_Toc196408359)

[Table S1. PCA standards used in this study and respective calibration mixture compositions 3](#_Toc196408360)

[Table S2. HPLC gradient program settings used for the initial evaluation of PCA behavior across different stationary phases 4](#_Toc196408361)

[Table S3. MS/MS optimization mixture compositions 4](#_Toc196408362)

[Table S4. Theoretical PCA concentrations of the interlaboratory test materials acquired from the European Union Reference Laboratory for Dioxins and PCBs in Feed and Food (EURL POPs) 5](#_Toc196408363)

[Table S5. Summary of LC-MS/MS results from analyzing the EURL POPs interlaboratory test materials 5](#_Toc196408364)

[Table S6. Summary of LC-MS/MS results from analyzing the certified reference material ERM-CE100 (fish tissue) from the Joint Research Centre of the European Commission 6](#_Toc196408365)

[Figure S1. Theoretical isobaric overlaps of [M+Cl]^-^ species for PCA-C_10–17_Cl_4-9_ homologues under low-resolution MS conditions 7](#_Toc196408366)

[Figure S2. Elution behavior of PCAs-C_n_Cl_5_ and PCAs-C_n_Cl_8_ on Phenyl-Hexyl (A) and Biphenyl (B) stationary phases under isocratic mobile phase conditions 8](#_Toc196408367)

[Figure S3. Mobile phase gradient types that were used for the final optimization of the PCA separation method 9](#_Toc196408368)

[Figure S4. The relative abundance of deprotonated and acetate adducts between different PCA homologue groups (expressed as the percentage of the total sum peak area of both adducts) 10](#_Toc196408369)

[Figure S5. The relative abundance of deprotonated and chloride adducts between different PCA homologue groups (expressed as the percentage of the total sum peak area of both adducts) 10](#_Toc196408370)

[Figure S6. Fragmentation behavior of acetate and chloride adducts of different PCA homologue groups 11](#_Toc196408371)

[Figure S7. Scanning ranges of selected SRM transitions (left) and cumulative overlap of SRM scans over time (right) 12](#_Toc196408372)

# S1. Summary of instrumental parameters for the optimization experiments

The stationary phase and gradient conditions were optimized using an LC-ESI-HRMS system (Dionex UltiMate 3000 UHPLC coupled to a Q Exactive Orbitrap MS) in full-MS mode. The optimization process involved three stages to identify the most effective stationary phase and gradient parameters.

First, the performance of six different columns was evaluated using nine generic gradient programs (Table S2). Based on these results, the two most promising columns were further tested under isocratic conditions with varying organic phase compositions to determine the optimal gradient range (Figure S1). Finally, both columns were fine-tuned under gradient conditions for optimal performance (Figure S2).

Ionization and fragmentation were optimized in two stages. In the first stage, standard mixtures of single-chain PCAs were prepared (Table S3). Two test solutions (1 ng/μL) were made in methanolic solutions containing either 0.025 mM ammonium chloride or 5 mM ammonium acetate. These mixtures were directly infused into a TSQ Altis Triple Quadrupole mass spectrometer at a 10 μL/min flow rate and evaluated using the automatic optimization wizard in TSQ Altis Tune Application 3.4. For each PCA-CxCly homologue group, the two to three most intense fragment ions were advanced to the following optimization stage.

In the second stage, previously identified fragments were further refined under LC conditions. Each single-chain mixture was injected into the LC-MS/MS system using the optimized gradient. SRM transitions were fine-tuned by testing collision energies ranging from 5 to 50 V.

# Table S1. PCA standards used in this study and respective calibration mixture compositions

| PCA single-chain standard | Concentration, ng/µL | Calibration mixture | | | | |
| --- | --- | --- | --- | --- | --- | --- |
|  |  | **A** | **B** | **C** | **D** |  |
| *Chloroparaffin* C10 50% Cl* | 10 | **x** |  |  |  |  |
| *Chloroparaffin* C11 50% Cl* | 10 | **x** |  |  |  |  |
| *Chloroparaffin* C12 45% Cl* | 10 | **x** |  |  |  |  |
| *Chloroparaffin* C13 43%** | 10 | **x** |  |  |  |  |
| *Chloroparaffin* C14 40%** | 10 | **x** |  |  |  |  |
| *Chloroparaffin* C15 39%** | 10 | **x** |  |  |  |  |
| *Chloroparaffin* C16 52%** | 10 | **x** |  |  |  |  |
| *Chloroparaffin* C17 43%** | 10 | **x** |  |  |  |  |
| *Chloroparaffin* C10 55% Cl* | 10 |  | **x** |  |  |  |
| *Chloroparaffin* C11 55% Cl* | 10 |  | **x** |  |  |  |
| *Chloroparaffin* C12 50% Cl* | 10 |  | **x** |  |  |  |
| *Chloroparaffin* C13 52%** | 10 |  | **x** |  |  |  |
| *Chloroparaffin* C14 53%** | 10 |  | **x** |  |  |  |
| *Chloroparaffin* C15 51%** | 10 |  | **x** |  |  |  |
| *Chloroparaffin* C16 53%** | 10 |  | **x** |  |  |  |
| *Chloroparaffin* C17 50%** | 10 |  | **x** |  |  |  |
| *Chloroparaffin* C10 60% Cl* | 10 |  |  | **x** |  |  |
| *Chloroparaffin* C11 61% Cl* | 10 |  |  | **x** |  |  |
| *Chloroparaffin* C12 55% Cl* | 10 |  |  | **x** |  |  |
| *Chloroparaffin* C13 60% Cl* | 10 |  |  | **x** |  |  |
| *Chloroparaffin* C14 55%** | 10 |  |  | **x** |  |  |
| *Chloroparaffin* C15 55%** | 10 |  |  | **x** |  |  |
| *Chloroparaffin* C16 71%** | 10 |  |  | **x** |  |  |
| *Chloroparaffin* C17 61%** | 10 |  |  | **x** |  |  |
| *Chloroparaffin* C10 65% Cl* | 10 |  |  |  | **x** |  |
| *Chloroparaffin* C11 65% Cl* | 10 |  |  |  | **x** |  |
| *Chloroparaffin* C12 65% Cl* | 10 |  |  |  | **x** |  |
| *Chloroparaffin* C13 65% Cl* | 10 |  |  |  | **x** |  |

* Commercial chain-length specific standard mixture from Dr. Ehrenstorfer (Germany).

** Chain-length specific standard mixture, which has been synthesized in the University of Hohenheim and obtained from the European Union Reference Laboratory for Halogenated Persistent Organic Pollutants in Feed and Food (EURL POPs).

# Table S2. HPLC gradient program settings used for the initial evaluation of PCA behavior across different stationary phases

| No. | Flow rate, mL/min | Mobile phase B at the start of gradient, % | Initial hold time, min | Time to reach %B=100%, min | Hold time, min |
| --- | --- | --- | --- | --- | --- |
| 1 | 0.250 | 10 | 1 | 5.0 | 12 |
| 2 |  |  |  | 7.5 |  |
| 3 |  |  |  | 10.0 |  |
| 4 |  | 30 |  | 5.0 |  |
| 5 |  |  |  | 7.5 |  |
| 6 |  |  |  | 10.0 |  |
| 7 |  | 60 |  | 5.0 |  |
| 8 |  |  |  | 7.5 |  |
| 9 |  |  |  | 10.0 |  |

# Table S3. MS/MS optimization mixture compositions

| Single-chain standard | MS/MS optimization mixture |
| --- | --- |
| *Chloroparaffin* C10 50.18% Cl | C10 MS/MS mixture, 10 ng/μL |
| *Chloroparaffin* C10 65.02% Cl |  |
| *Chloroparaffin* C11 45.50% Cl | C11 MS/MS mixture, 10 ng/μL |
| *Chloroparaffin* C11 60.53% Cl |  |
| *Chloroparaffin* C12 45.32% Cl | C12 MS/MS mixture, 10 ng/μL |
| *Chloroparaffin* C12 65.08% Cl |  |
| *Chloroparaffin* C13 43.07% | C13 MS/MS mixture, 10 ng/μL |
| *Chloroparaffin* C13 65.18% Cl |  |
| *Chloroparaffin* C14 40.36% | C14 MS/MS mixture, 10 ng/μL |
| *Chloroparaffin* C14 55.09% |  |
| *Chloroparaffin* C15 39.49% | C15 MS/MS mixture, 10 ng/μL |
| *Chloroparaffin* C15 55.03% |  |
| *Chloroparaffin* C16 51.62% | C16 MS/MS mixture, 10 ng/μL |
| *Chloroparaffin* C16 70.55% |  |
| *Chloroparaffin* C17 43.05% | C17 MS/MS mixture, 10 ng/μL |
| *Chloroparaffin* C17 61% |  |

# Table S4. Theoretical PCA concentrations of the interlaboratory test materials acquired from the European Union Reference Laboratory for Dioxins and PCBs in Feed and Food (EURL POPs)

| Name | Code | Matrix | Weight for the analysis, g | Theoretical concentration, μg/kg | | |
| --- | --- | --- | --- | --- | --- | --- |
|  |  |  |  | PCA-C_10-13_ | PCA-C_14-17_ | PCA-C_10-17_ |
| Lard B | LB | Lard | 1,0 | 69 | 56 | 125 |
| Lard D | LD | Lard | 1,0 | 31 | 94 | 125 |
| Lard E | LE | Lard | 1,0 | 149 | 121 | 270 |
| Coconut D | CD | Coconut fat | 1,0 | 120 | 180 | 300 |
| Coconut C | CC | Coconut fat | 1,0 | -^[[1]](#footnote-1)^ | 300 | - |
| Coconut B | CB | Coconut fat | 1,0 | 300 | - | - |

Table S5. Summary of LC-MS/MS results from analyzing the EURL POPs interlaboratory test materials

| Code | PCA-C_10-13_ | | | PCA-C_14-17_ | | | PCA-C_10-17_ | | |
| --- | --- | --- | --- | --- | --- | --- | --- | --- | --- |
|  | Res.^[[2]](#footnote-2)^, μg/kg | Rec.^[[3]](#footnote-3)^, % | Z-Score^[[4]](#footnote-4)^ | Res., μg/kg | Rec., % | Z-Score | Res., μg/kg | Rec., % | Z-Score |
| LB1 | 43 | 66 | -1.3 | 38 | 68 | -1.3 | 81 | 67 | -1.3 |
| LB2 | 46 | 70 | -1.2 | 89 | 159 | 2.3 | 135 | 111 | 0.5 |
| LB3 | 48 | 74 | -1.0 | 72 | 129 | 1.1 | 120 | 99 | 0.0 |
| LD1 | 28 | 90 | -0.4 | 80 | 86 | -0.6 | 108 | 87 | -0.5 |
| LD2 | 29 | 92 | -0.3 | 70 | 75 | -1.0 | 99 | 79 | -0.8 |
| LD3 | 22 | 70 | -1.2 | 71 | 75 | -1.0 | 92 | 74 | -1.0 |
| LE1 | 83 | 56 | -1.8 | 104 | 86 | -0.6 | 187 | 69 | -1.2 |
| LE2 | 82 | 55 | -1.8 | 115 | 95 | -0.2 | 197 | 73 | -1.1 |
| LE3 | 95 | 64 | -1.4 | 105 | 87 | -0.5 | 201 | 74 | -1.0 |
| CD1 | 83 | 69 | -1.2 | 250 | 139 | 1.5 | 333 | 111 | 0.4 |
| CD2 | 70 | 58 | -1.7 | 194 | 108 | 0.3 | 264 | 88 | -0.5 |
| CD3 | 101 | 84 | -0.6 | 283 | 157 | 2.3 | 384 | 128 | 1.1 |
| CC1 | 20 | - | - | 351 | 117 | 0.7 | 371 | - | - |
| CC2 | 23 | - | - | 285 | 95 | -0.2 | 308 | - | - |
| CC3 | 17 | - | - | 271 | 90 | -0.4 | 288 | - | - |
| CB1 | 277 | 92 | -0.3 | 73 | - | - | 350 | - | - |
| CB2 | 348 | 116 | 0.6 | 81 | - | - | 429 | - | - |
| CB3 | 283 | 94 | -0.2 | 42 | - | - | 325 | - | - |

# Table S6. Summary of LC-MS/MS results from analyzing the certified reference material ERM-CE100 (fish tissue) from the Joint Research Centre of the European Commission

| Sample | PCA-C_10-13_, μg/kg | Certified value ± uncertainty, μg/kg | Recovery, % |
| --- | --- | --- | --- |
| #1-ERM-CE100 | 25.8 | 31±9 | 83% |
| #2-ERM-CE100 | 24.5 | 31±9 | 79% |
| #3-ERM-CE100 | 33.7 | 31±9 | 109% |
|  |  |  |  |
| Sample | **PCA-C_14-17_, μg/kg** | **Certified value ± uncertainty, μg/kg** | **Recovery, %** |
| #1-ERM-CE100 | 44.8 | 44±17 | 102% |
| #2-ERM-CE100 | 27.0 | 44±17 | 61% |
| #3-ERM-CE100 | 52.3 | 44±17 | 119% |

# Figure S1. Theoretical isobaric overlaps of [M+Cl]^-^ species for PCA-C_10–17_Cl_4-9_ homologues under low-resolution MS conditions


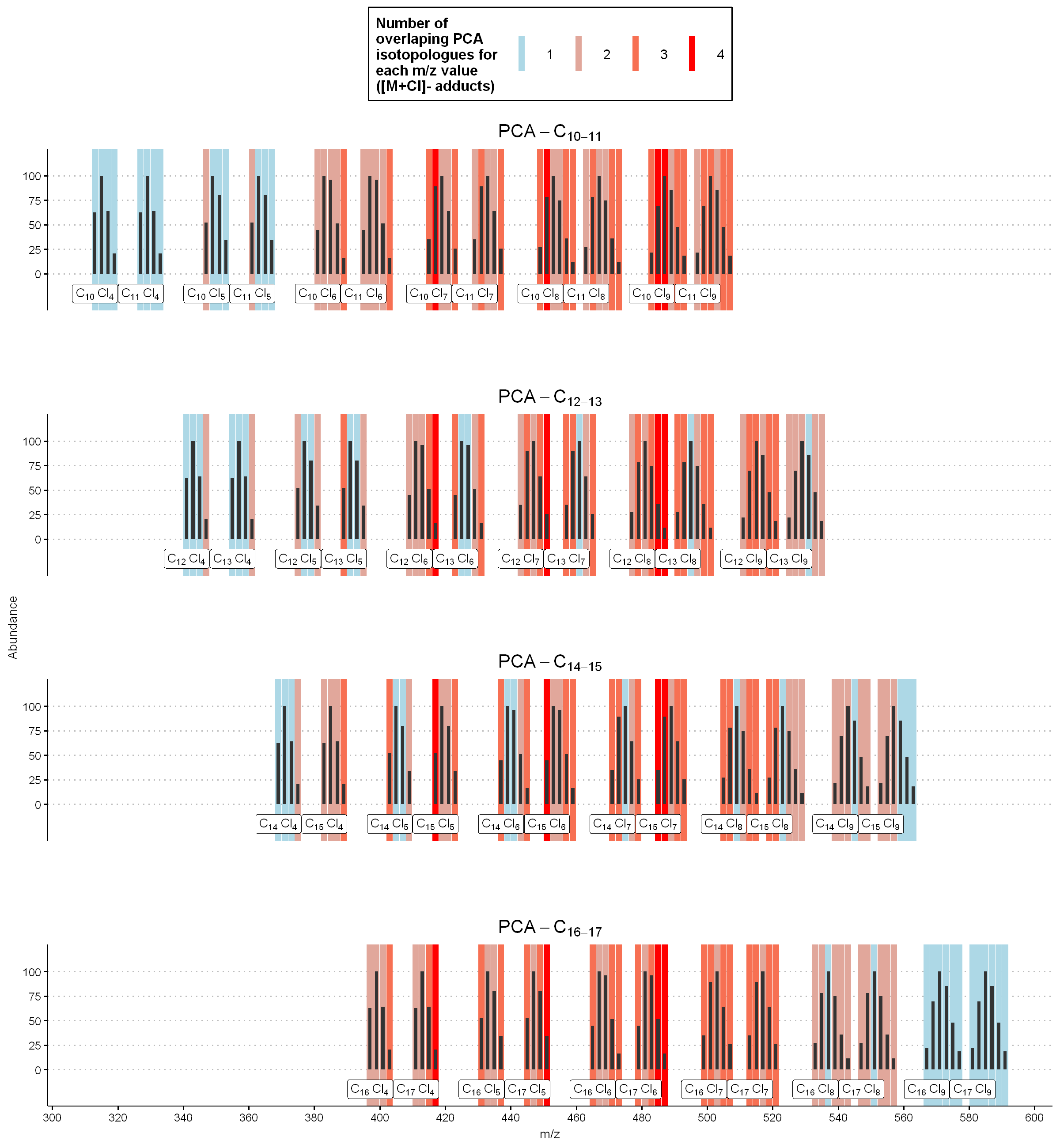


# Figure S2. Elution behavior of PCAs-C_n_Cl_5_ and PCAs-C_n_Cl_8_ on Phenyl-Hexyl (A) and Biphenyl (B) stationary phases under isocratic mobile phase conditions


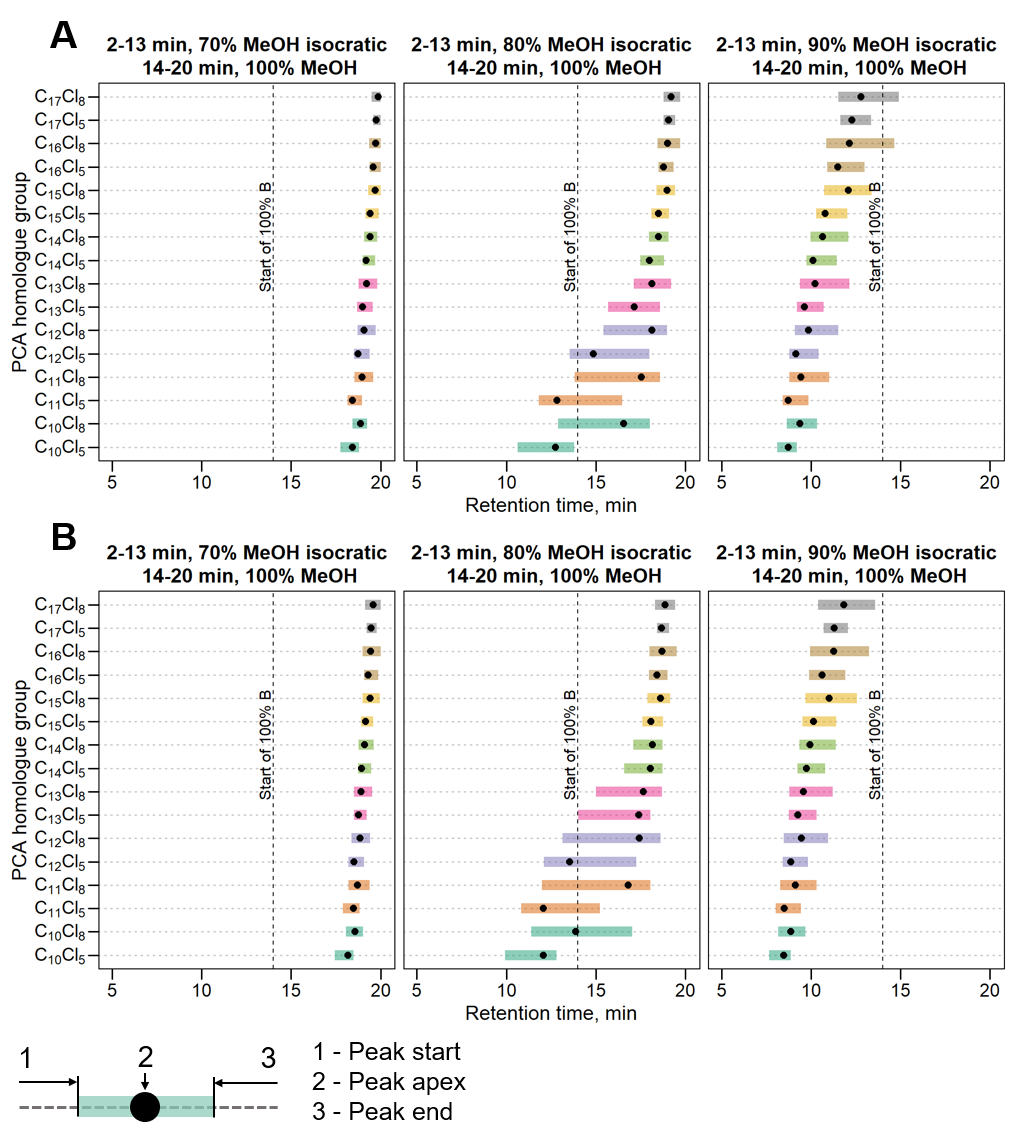


# Figure S3. Mobile phase gradient types that were used for the final optimization of the PCA separation method


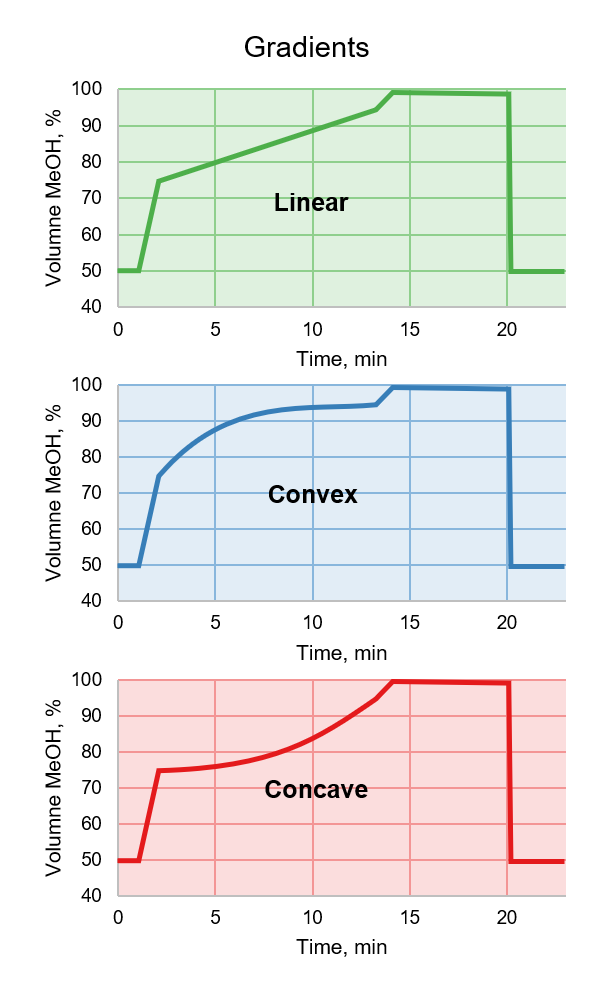


# Figure S4. The relative abundance of deprotonated and acetate adducts between different PCA homologue groups (expressed as the percentage of the total sum peak area of both adducts)


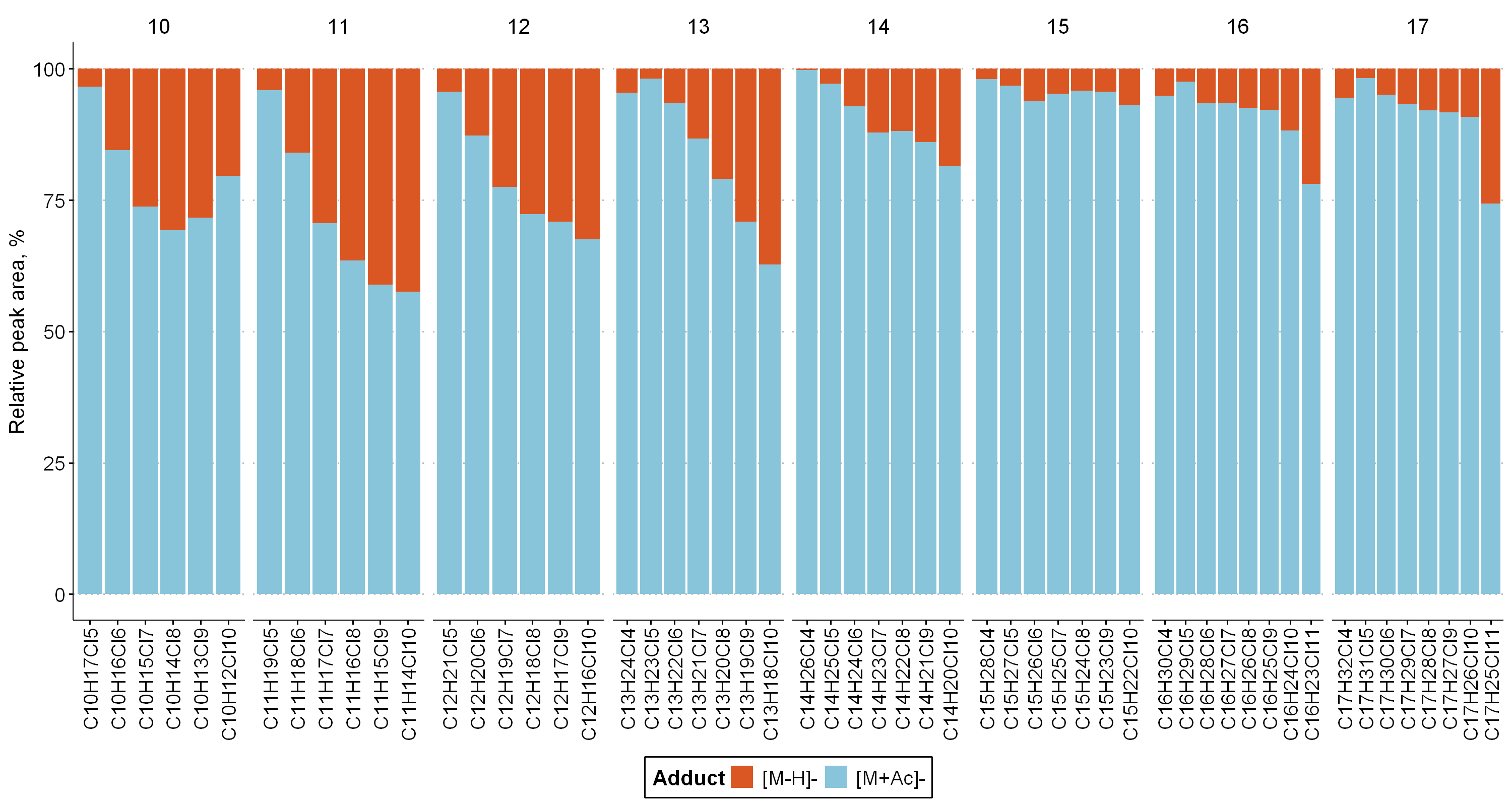


# Figure S5. The relative abundance of deprotonated and chloride adducts between different PCA homologue groups (expressed as the percentage of the total sum peak area of both adducts)


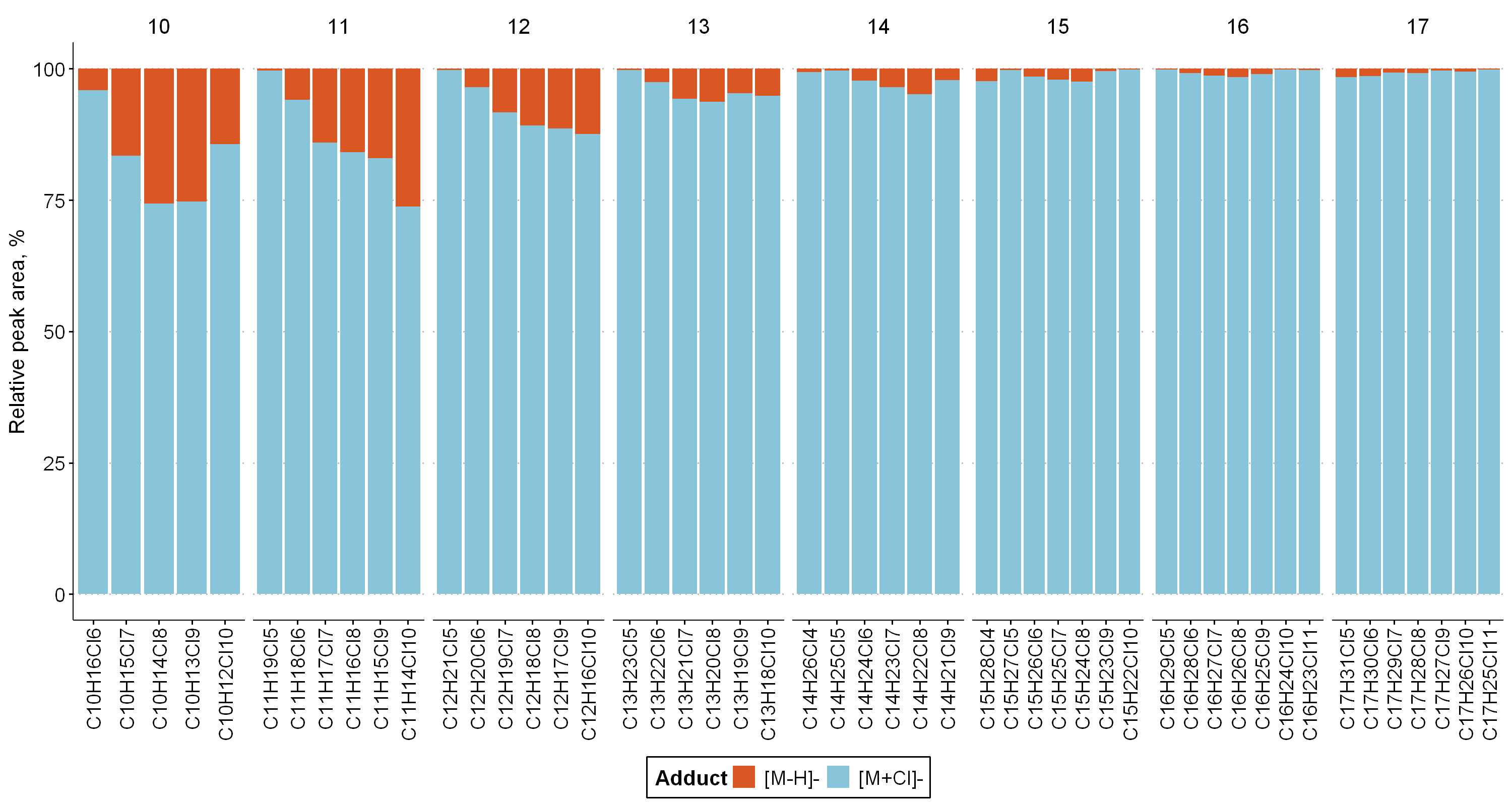


# Figure S6. Fragmentation behavior of acetate and chloride adducts of different PCA homologue groups


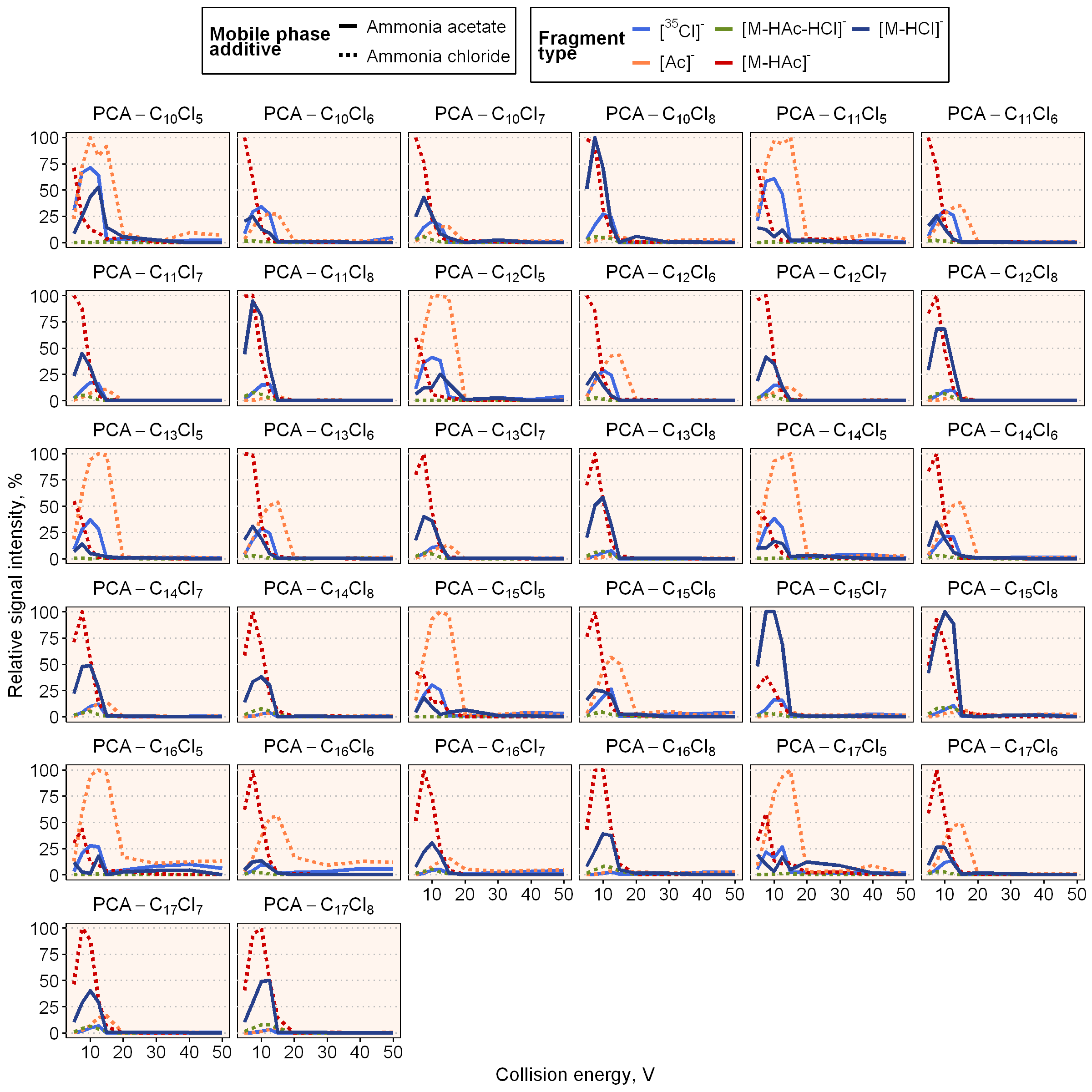


# Figure S7. Scanning ranges of selected SRM transitions (left) and cumulative overlap of SRM scans over time (right)


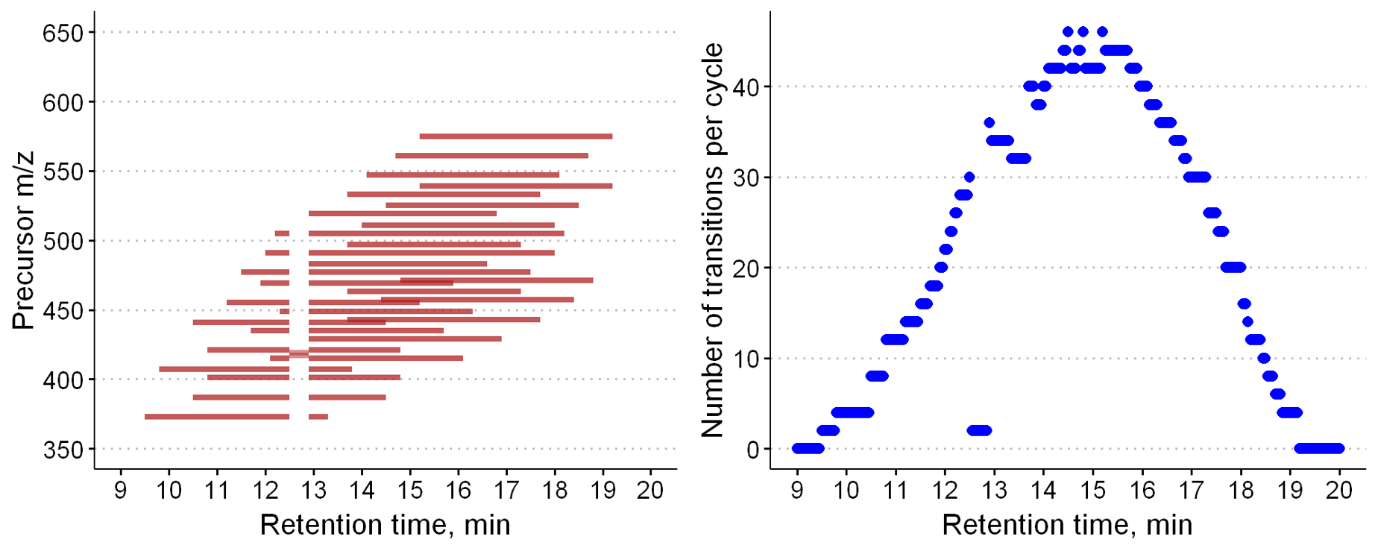


1. No theoretical value was assigned in the interlaboratory study. [↑](#footnote-ref-1)
2. Res. - measured result from analysis. [↑](#footnote-ref-2)
3. Rec. – recovery (measured value / theoretical value * 100%). [↑](#footnote-ref-3)
4. Z-scores were calculated according to the methodology used in the interlaboratory study, where the fitness-for-purpose standard deviation (σ_p_) for proficiency assessment was established at 25%. [↑](#footnote-ref-4)
